# Planar Four-Membered Diboron Actinide Compound with Double Möbius Aromaticity

**DOI:** 10.1021/jacs.3c00907

**Published:** 2023-03-28

**Authors:** Xuhui Lin, Wei Wu, Yirong Mo

**Affiliations:** † School of Chemistry, Southwest Jiaotong University, Chengdu, Sichuan 610031, China; ‡ The State Key Laboratory of Physical Chemistry of Solid Surfaces, iChEM, Fujian Provincial Key Laboratory of Theoretical and Computational Chemistry and College of Chemistry and Chemical Engineering, Xiamen University, Xiamen, Fujian 361005, China; § Department of Nanoscience, Joint School of Nanoscience and Nanoengineering, University of North Carolina at Greensboro, Greensboro, North Carolina 27401, United States

## Abstract

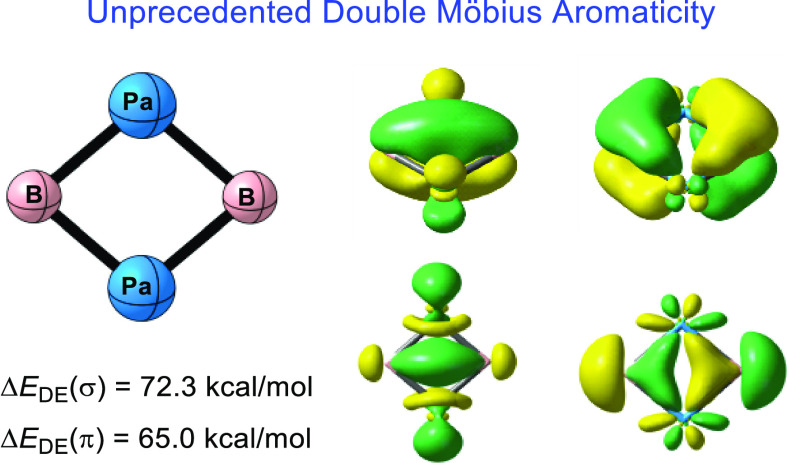

The Möbius rule predicts that a planar four-membered metallacycle can be aromatic with four mobile electrons, but such a simple ring has escaped recognition because it usually favors Hückel anti-aromaticity. Here, we report that a quasi-square four-membered actinide compound (Pa_2_B_2_) is doubly Möbius aromatic. Chemical bonding analyses reveal that this diboron protactinium molecule has four delocalized π electrons in addition to four delocalized σ electrons, satisfying the 4n Möbius rule for both σ and π components. Energetically, the block-localized wavefunction method, which is the simplest variant of ab initio valence bond theory, shows that the delocalization energy for the π and σ electrons reaches up to 65.0 and 72.3 kcal/mol, respectively, while the extra cyclic resonance energy (ECRE) amounts to 45 kcal/mol. The large positive ECRE values strongly confirm the unprecedented double Möbius aromaticity in Pa_2_B_2_. We anticipate that this new type of aromatic molecule can enrich the concept of Möbius aromaticity and open a new avenue for actinide compounds.

## Introduction

**Figure 1:**
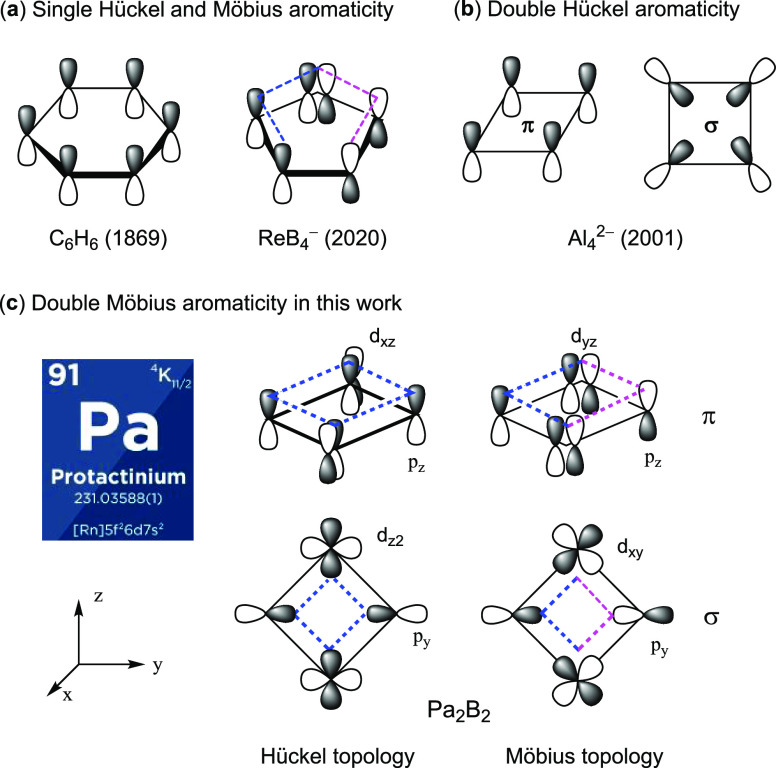
(a) Single Hückel and Möbius aromaticity in benzene and ReB_4_^–^ with the most representative molecular orbitals, (b) double Hückel aromaticity in Al_4_^2–^, and (c) unprecedented double Möbius aromaticity in Pa_2_B_2_ proposed in this work.

Aromaticity is one of the central concepts in chemistry that accounts for the unusual stability of conjugated molecules, clusters, and materials.^1−3^ The term “aromaticity” was originally bestowed on benzene and related organic molecules featuring cyclic delocalization of π_p_ electrons (Figure 1a),^4^ and later, the concept was extended to metallaaromaticity by Thorn and Hoffmann for metallacycles.^5^ In this regard, the bonding picture is changed from π_p–p_ to π_d–p_ conjugated systems due to the participation of *d* orbitals from transition metals.^6−8^ Furthermore, the introduction of transition metals also makes it possible for planar metallacycles to exhibit the novel Craig-Möbius aromaticity,^9−11^ though it usually refers to annulenes with a twist molecular topology that resemble a Möbius strip.^12−14^ Different from the conventional 4n + 2 Hückel aromaticity, Möbius aromaticity allows for an aromatic system with 4n mobile electrons due to the phase change of overlapping π orbitals.^15^

In the past several decades, numerous organometallic, all-metal, and semi-metal species have been recognized to exhibit single or multiple Hückel aromaticity.^16−20^ In particular, Wang and co-workers characterized the isolated Al_4_^2–^ (Figure 1b) and related clusters as doubly aromatic arising from σ and π orbitals.^16^ Very recently, a three-membered 4*f*-metallaaromatic molecule PrB_2_ was reported by Wang et al.^21^ Unlike the much familiar Hückel aromaticity, however, only a few Craig-Möbius aromatic systems including planar bicyclic osmapentalynes and metallaborocycles (ReB_4_^–^, Figure 1a) have been well identified both experimentally and theoretically.^22−24^ As a consequence, the knowledge of Craig-Möbius aromaticity lags far behind the Hückel aromaticity. For instance, it has remained restricted to the π system in mono-metallacycles. According to the Hückel rule, a four-membered ring with four mobile electrons in the ground state (such as cyclobutadiene) should be antiaromatic,^25^ while it can reverse to be aromatic based on the Möbius rule. However, realization of such π-, σ-, and even doubly aromatic four-membered rings is still elusive because four-membered rings usually exhibit anti-aromaticity or aromaticity only in their excited states as predicted by Baird’s rules.^26^

Actinides are a unique group of elements in terms of chemical bonding. Since the 5*f* atomic orbitals (AOs) are rather contracted and 6*d* AOs are energetically high, the bonding involving actinides is predicted to be weak and localized.^27−29^ In other words, actinides are rarely found to participate in strong and delocalized aromaticity, typically with a light 2*p* element.^30^ Remarkably, a unique σ-aromatic thorium–thorium bond was recently observed in the crystalline tri-thorium cluster,^31^ and the claim of such σ-aromaticity has been authenticated by the ab initio valence bond (VB) method.^32^ Besides, metal clusters [Th@Bi_12_]^4–^ and U_4_(NH)_4_ have also been found to exhibit single and double aromaticity, respectively.^33,34^ It is worthy to note that Kiplinger and co-workers prepared actinide 2-metallabiphenylenes containing an aromatic benzene ring and an antiaromatic cyclobutadiene ring.^35^

Protactinium (Pa, [Rn]5*f*^2^6*d*^1^7*s*^2^) is the first actinide to contain 5*f* electrons, exhibiting properties intermediate between thorium and uranium.^36^ The unique electronic structure of Pa makes it essential as a stepping stone to study the periodic properties of early actinide elements. Because Pa is highly radioactive and toxic, it puzzled chemists for a long time before finding its true place in fundamental research. This becomes increasingly clear with the development of modern computational chemistry, suggesting that protactinium still has more to contribute to the understanding of the electronic structures and bonding behaviors of 5*f* elements. For example, Pa can be used to design rational actinide compounds with light 2*p* elements.^37,38^ As an electron-deficient element, boron has higher 2*p* orbitals compared to carbon and nitrogen,^39^ which can form efficient bonding with energetically high-lying 5*f* and 6*d* orbitals.

Given that double Craig-Möbius aromaticity has escaped the recognition so far, typically in four-membered actinide rings, here we propose an actinide compound Pa_2_B_2_, which exhibits unprecedented double Möbius aromaticity. Specifically, the novel Pa_2_B_2_ possesses four delocalized π electrons and four delocalized σ electrons (Figure 1c), including two π or σ electrons in an orbital of Möbius topology.

## Methods

Geometry optimizations were performed by Gaussian 16^40^ with the PBE0 functional,^41^ in which a small-core fully relativistic effective core potential (ECP60MDF)^42^ and associated segmented valence basis sets were adopted for Pa and the def2-TZVP basis set was used for boron. We also employed confirmative geometry optimization with second-order Douglas–Kroll–Hess (DKH2) relativistic Hamiltonian,^43,44^ PBE0 functional, SARC-DKH2 basis set^45^ for Pa, and def2-TZVP basis set for B. All calculations were augmented with Grimme’s D3 dispersion corrections.^46^ In this paper, the data listed in the following are taken from PBE0-DKH2/SARC-DKH2 without special mention, while the similar results by PBE0/ECP60MDF are compiled in the Supporting Information for comparison. It should be noted that we also performed calculations with the DKH4 correction and obtained nearly identical results to those with the DKH2 correction.

The BLW calculations were performed with the in-house version of GAMESS^47^ and XMVB softwares.^48,49^ The QTAIM and AdNDP analysis were obtained with the Multiwfn package.^50^ The CYLview^51^ and VMD^52^ programs were used for the visualization of structures and molecular orbitals, respectively.

## Results and Discussion

**Figure 2:**
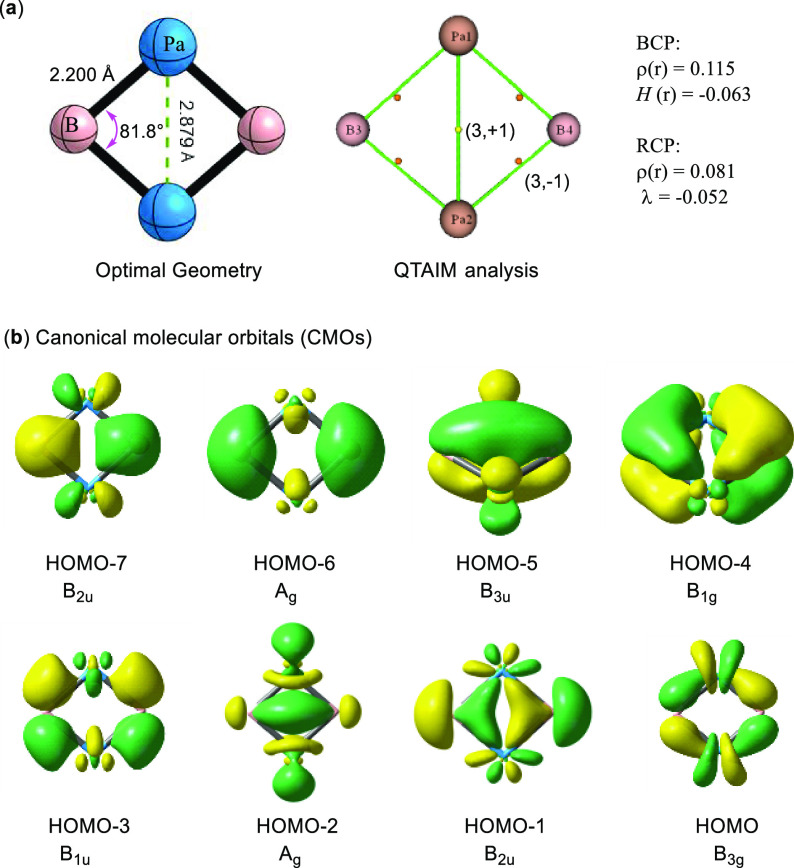
(a) Optimal geometry and QTAIM topological analyses, including the electron density ρ(*r*), electron energy density *H*(*r*), and electron density curvature (λ) at BCP (3, −1) or RCP (3, +1); (b) valence canonical molecular orbitals (CMOs) with an isovalue = 0.05 a.u.

The most stable structure is found to have a near square planar geometry (as shown in Figure 2a) with *D*_2h_ symmetry. The four Pa–B bonds are identical to each other and the bond distance (2.200 Å) is much shorter than the sum of the covalent radii of Pa (2.00 Å) and B (0.84 Å),^53^ indicating multiple bonding characters in Pa–B bonds. This is also evidenced by the Mayer bond index (1.724) for Pa–B bonds. Surprisingly, the distance between two protactiniums shortens to 2.879 Å, in consistent with the predicted double Pa = Pa bond with the additive covalent radii of 2.86 Å.^54^ However, the Mayer bond index for the Pa = Pa bond is 2.330, indicating that the four-membered ring of Pa_2_B_2_ may possess cyclic electron delocalization. Moreover, the bond angles for Pa–B–Pa and B–Pa–B are 81.8 and 98.2°, respectively. To ascertain the thermal stability of Pa_2_B_2_, the Born–Oppenheimer molecular dynamics (BOMD) was performed at 300, 500, and 700 K (see Figure S2), and we found that Pa_2_B_2_ is very stable at room temperature and even high temperatures.

To better understand the quasi-square structure and bonding pattern in Pa_2_B_2_, we first employed the quantum theory of atoms in molecules (QTAIM) method^55,56^ to search for bond or ring critical points among bound atoms. As expected, we found four identical bond critical points (BCPs) between each Pa and B atoms, referring to the four localized Pa–B covalent bonds which are supported by the large electron density (0.115) and negative energy densities (−0.063) at BCPs. In sharp contrast, the identification of a ring critical bond (RCP) in the center of square Pa_2_B_2_ rather than a BCP between two Pa atoms suggests that the short Pa–Pa distance originates from the cyclic electron delocalization. It has been acknowledged that a positive electron density together with a negative electron density curvature at the RCP can be used as a rough measure for aromaticity, and the present calculated values for Pa_2_B_2_ are 0.08 and −0.05, respectively. This finding indicates that the four-membered Pa_2_B_2_ system seems to exhibit unprecedented aromaticity.

We continued to study the Kohn–Sham CMOs for Pa_2_B_2_ and quantified their composition by the natural atomic orbital (NAO) analysis^57^ (see details in Table S1). Since there are five valence electrons in Pa and three valence electrons in B, the total valence CMOs for Pa_2_B_2_ are eight. Among them (Figure 2b), we found two delocalized π orbitals, i.e., HOMO-4 and HOMO-5, which are well consistent with the schematical π orbitals in Figure 1c. HOMO-5 has a classical Hückel topology, consisting of the radial *d_xz_* orbitals of metal and *p_z_* orbitals from boron. On the contrary, HOMO-4 falls into a novel Möbius topology with a phase change induced by the tangential *d_yz_* orbitals. It is worth noting that the contributions of 5*f* atomic orbitals to HOMO-4 and HOMO-5 are 18 and 10%, respectively. A similar delocalization effect has also been observed in the σ framework, containing one delocalized σ orbital in Hückel topology (HOMO-2) and the other in Möbius topology (HOMO-1). In agreement with Figure 1c, HOMO-2 mainly comes from the *p_y_* orbitals of boron and the *d*_*z*2_ of metals, while the *d_xy_* orbitals of Pa along with the *p_y_* orbitals of B are used to construct HOMO-1. Particularly, the contribution of 5*f* orbitals to HOMO-2 reaches up to 34%, indicating the important role of 5*f* AOs in the formation of delocalized CMOs.

Thus, our examinations of the structural parameters and CMOs suggested that Pa_2_B_2_ exhibits unprecedented double Möbius aromaticity. On one hand, there are two independent delocalized systems each containing four electrons, satisfying the 4n Möbius rule and giving rise to double σ + π aromaticity. On the other hand, the four-membered ring possesses a quasi-square structure with an identical Pa–B bond distance, resulting from the delocalization of both σ and π electrons, exactly as expected for aromatic molecules.

**Figure 3:**
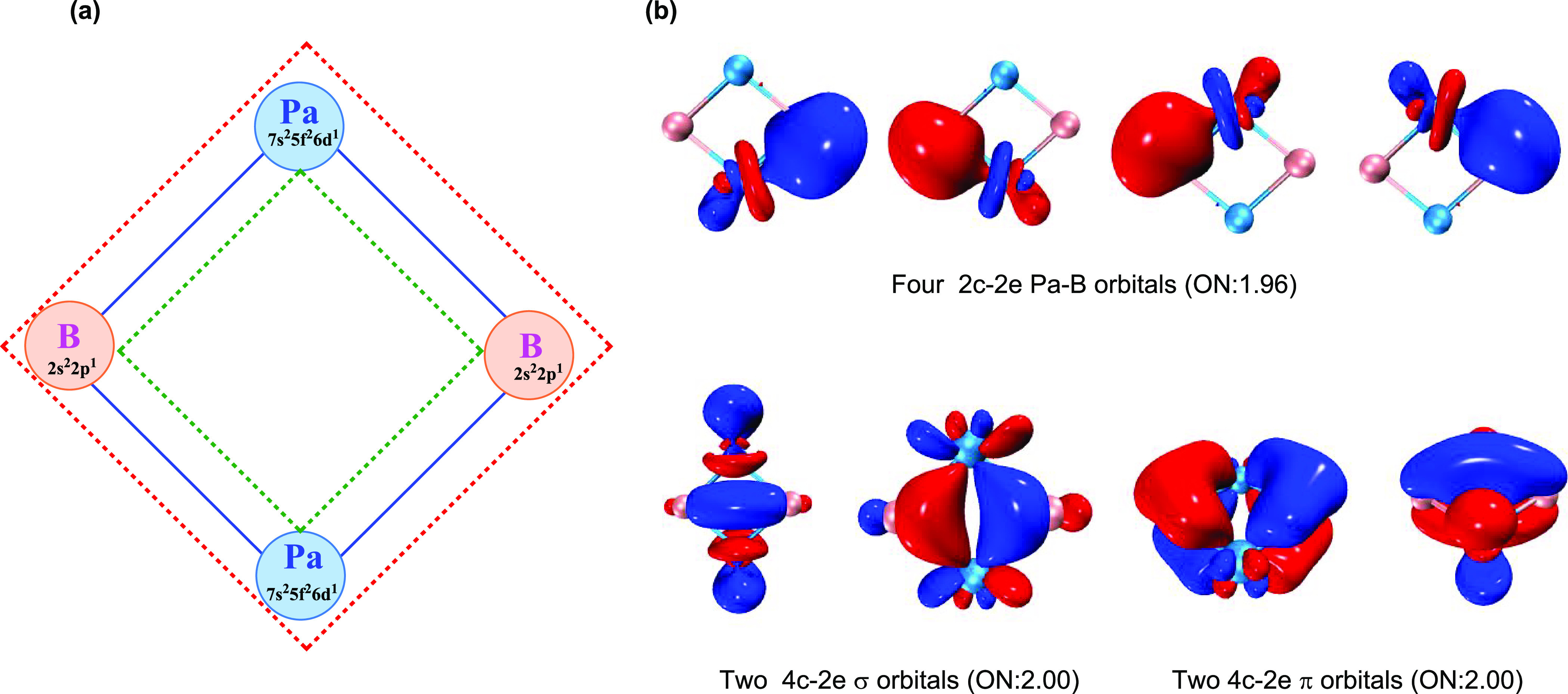
(a) Proposed bonding pattern, including four localized Pa–B covalent bond (solid blue line), two delocalized σ (inner dashed green line), and two delocalized π (outer dashed red line) bonds; (b) AdNDP analysis showing four localized 2c–2e Pa–B bonds and four delocalized 4c–2e aromatic bonds along with their occupation numbers (ONs).

We also analyzed the bonding nature in the Pa_2_B_2_ system by using the adaptive natural density partitioning (AdNDP) method,^58^ which has been widely used to assess aromaticity in transition metal compounds. As shown in Figure 3b, apart from four identical localized 2c–2e Pa–B bonds, there are four delocalized 4c–2e bonds, including two σ bonds and two π bonds. The observation of a fully delocalized 4c–2e bonding pattern further authenticates the double Möbius aromaticity in the planar four-membered ring. In brief, the total 16 valence electrons form three kinds of chemical bonds, i.e., four identical localized Pa–B σ covalent bonds, two delocalized σ bonds, and two delocalized π bonds (Figure 3a).

Since the widely used nuclear-independent chemical shift (NICS)^59^ and related ring current criteria are not suitable to assess the aromaticity in small rings and heavy metal compounds,^60−62^ we resorted to ab initio valence bond (VB) theory^63−65^ to seek an improved understanding of the aromaticity in this novel Pa_2_B_2_ molecule from the energetic point of view. Ultimately, aromaticity is equivalent to the extra stability. The ab initio VB method can construct wave functions for Lewis (resonance or electron-localized) structures with strictly localized atomic or fragmental orbitals and estimate the energy change due to electron delocalization. Notably, the block-localized wavefunction (BLW) method which is the simplest variant of ab initio VB theory can define and optimize a particular resonance state at the DFT level.^66−69^

In the BLW computations, each atom in Pa_2_B_2_ was treated as a block, and thus, pure AOs were used to construct strictly block-localized wavefunctions. Besides the core orbitals, the eight valence CMOs can be divided into three groups as we discussed above, i.e., four localized Pa–B σ orbitals and two delocalized σ and two delocalized π orbitals. In the delocalized state (Ψ_del_) from the regular DFT computations, all eight CMOs are delocalized with contributions from all basis functions. To explore the electron delocalization effect among the above three kinds of CMOs individually, we constructed localized states step by step (see details in Supporting Information). First, the four localized Pa–B σ orbitals were strictly localized on corresponding diatoms and the remaining MOs were fully delocalized. Therefore, the energy change (Δ*E*_cov_) from this localized state Ψ_loc_^cov^ to the delocalized Ψ_del_ reflects the electron delocalization among the four Pa–B covalent bonds. The insignificant value of Δ*E*_cov_ (3.5 kcal/mol) is consistent with the previous conclusion that the four Pa–B σ bonds are essentially localized. Second, we established strictly σ- and π-localized states (Ψ_loc_^σ^ and Ψ_loc_^π^) by further localizing two σ and two π orbitals on two adjacent Pa and B atoms separately. Finally, all the eight CMOs were localized to construct the completely localized state (Ψ_loc_^tot^). In these computations, energy differences of σ-, π-, or totally localized states with reference to Ψ_loc_^cov^ measure the electron delocalization induced by σ and π orbitals and all orbitals, respectively. The electron delocalization energies for the σ and π components are 72.3 and 65.0 kcal/mol, while the total electron delocalization energy amounts to 160.9 kcal/mol. The much high electron delocalization energies again confirm that the σ and π orbitals are highly delocalized, while the total delocalization energy is higher than the sum of individual delocalization energies due to the coupling effect.

**Figure 4:**
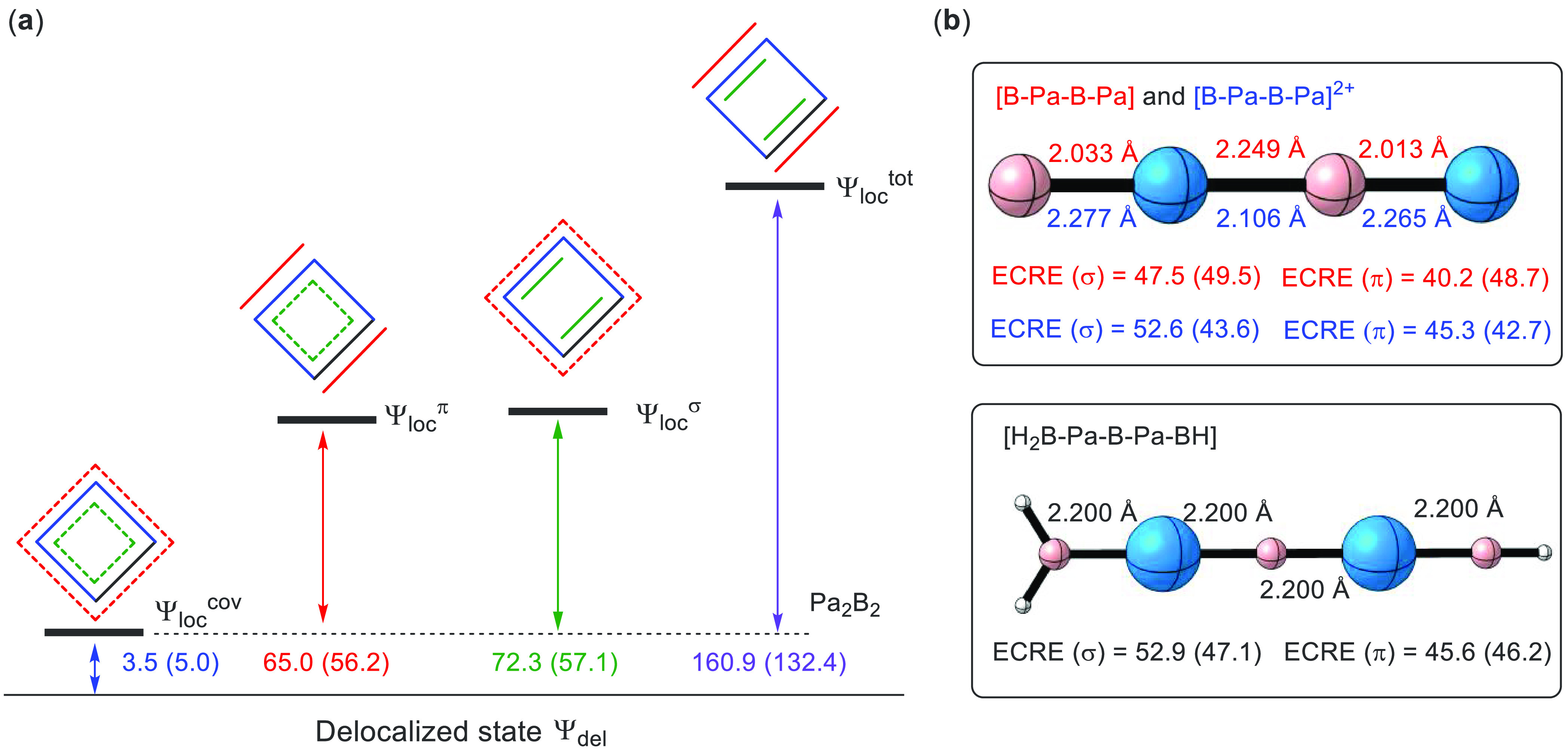
(a) Electron delocalization (resonance) energies (in kcal/mol) among the four Pa–B covalent bonds (in blue), two delocalized 4c–2e π bonds (in red), two delocalized 4c–2e σ bonds (in green), and all bonds; (b) evaluated extra cyclic resonance energy (ECRE, in kcal/mol) with acyclic Pa_2_B_2_ (red data), [Pa_2_B_2_]^2+^(blue data), and [H_2_B-Pa-B-Pa-BH] (black data) as reference. All the delocalization energies are obtained at the standard PBE0 level, while the results with DKH2 corrections are placed in the brackets.

Alternatively, the basis functions for planar Pa_2_B_2_ can be divided into the σ and π components. Therefore, we could use the BLW method to re-construct the Ψ_loc_^π^ state by only localizing the π electrons but with all σ electrons delocalizing over the whole system. It should be noted that we were unable to obtain the Ψ_loc_^σ^ state with the same strategy because the concerned delocalized σ orbitals share the same Pa atomic orbitals with localized Pa–B σ orbitals. The computed delocalization energy for the π system is 61.0 kcal/mol at the standard PBE0 level, which is comparable to 65.0 kcal/mol derived in Figure 4. Moreover, we also re-optimize this π-localized state, in which the two Pa–B bonds with π electrons shorten to 2.157 Å but the other two Pa–B bonds elongate to 2.357 Å, in agreement with the predicted single and double Pa–B bonds.^53^ Furthermore, the adiabatic delocalization energy between the optimal delocalized state and the optimal Ψ_loc_^π^ state is 53.5 kcal/mol.

However, we note that aromaticity refers to the “extra” stability in a cyclic system with reference to non-cyclic systems. The above electron delocalization energies cannot be simply used to justify the aromaticity in Pa_2_B_2_. In this regard, ECRE, defined as the delocalization energy difference between a cyclic compound and its appropriate acyclic reference, is more suitable for assessing the aromaticity.^70,71^ Specifically, a positive ECRE measures the magnitude of aromaticity, whereas negative ECRE corresponds to an antiaromatic system. The ECRE for a nonaromatic system thus should be around zero.

Herein, linear neutral Pa_2_B_2_ with *C*_∞v_ symmetry is considered as the acyclic reference to evaluate ECRE (Figure 4b). Because one Pa–B bond is broken in linear Pa_2_B_2_ compared to cyclic analogue, dicationic linear [Pa_2_B_2_]^2+^ is also used to obtain ECRE for comparison. For both references, we re-optimized their geometries at the *C*_∞v_ symmetry, though the optimal geometries are not necessarily the global minima. Besides, we also evaluated the ECREs with non-optimal linear Pa_2_B_2_ and [Pa_2_B_2_]^2+^ (see Figure S5), in which Pa–B bond distances are identical to that in the cyclic Pa_2_B_2_. As expected, the optimal and non-optimal linear Pa_2_B_2_ are much less stable than the cyclic Pa_2_B_2_ by 111.6 and 141.9 kcal/mol, respectively. From Figure 4b, one can see that the ECREs for both σ and π systems are about 45 kcal/mol, confirming again that the four-membered Pa_2_B_2_ ring is doubly Möbius aromatic. It should be noted that the above linear Pa_2_B_2_ and [Pa_2_B_2_]^2+^ both contain three Pa–B bonds, whereas the cyclic Pa_2_B_2_ has four bonds. Therefore, we considered another acyclic reference [H_2_B-Pa-B-Pa-BH] with four Pa–B bonds, where one hydrogen atom and BH_2_ group are added to the terminal B and Pa atoms, respectively. The calculated ECREs for the σ and π systems are comparable to those for linear Pa_2_B_2_, further confirming the double aromaticity in cyclic Pa_2_B_2_.

Although the synthesis of Pa_2_B_2_ is expected to be a challenge as Pa is rare, highly radioactive, and toxic, the significance of the present computational work lies in the elucidation of the unique bonding pattern in Pa_2_B_2_, which enriches the concept of aromaticity and opens a new avenue for actinide compounds. Furthermore, the double Möbius aromaticity may be observed in other four-membered compounds M_2_B_2_, where M refers to the protactinium’s transition metal homologues such as Nb and Ta.

## Conclusions

In this work, we performed an extensive computational study for the diboron protactinium compound (Pa_2_B_2_), which was found to be a planar ring with a quasi-square geometry. Chemical bonding analyses, including QTAIM, NBO, and AdNDP methods, revealed that this novel four-membered ring is doubly Möbius aromatic in the ground state, containing four delocalized σ and four delocalized π electrons. More importantly, the BLW method was employed to evaluate the electron delocalization energy and corresponding ECREs. The BLW results show that both the σ and π frameworks are highly delocalized as the computed delocalization energies reach up to 72.3 and 65.0 kcal/mol, respectively. Besides, the positive ECRE (ca. 45 kcal/mol for both σ and π components) strongly authenticates the double Möbius aromaticity in Pa_2_B_2_. Our study proved for the first time that σ- and π-Möbius aromaticity can co-exist in a planar four-membered ring.

## Supporting Information Available

Details on the BLW method and the construction of BLWs for Pa_2_B_2_, orbital composition analysis by the NAO method, BOMD analysis, CMOs and AdNDP analyses at the standard PBE0/ECP60MDF level, ECREs for the reference linear Pa_2_B_2_, and xyz coordinates of all studied structures (PDF)
