# Supplementary material for: Planar Four-Membered Diboron Actinide Compound with Double Möbius Aromaticity
Source: J Am Chem Soc. 2023 Mar 28;145(14):8107–13. doi: 10.1021/jacs.3c00907 (PMC10103132; doi:10.1021/jacs.3c00907)
Supplement: Supplementary file 1 — ja3c00907_si_001.pdf [file ja3c00907_si_001.pdf]

Supporting Information for

Planar Four-Membered Diboron Actinide Compound with Double Möbius Aromaticity

Xuhui Lin*[1], Wei Wu*[2] and Yirong Mo*[3]

[1] School of Chemistry, Southwest Jiaotong University, Chengdu, Sichuan 610031, China.

[2] The State Key Laboratory of Physical Chemistry of Solid Surfaces, iChEM, Fujian Provincial Key Laboratory of Theoretical and Computational Chemistry and College of Chemistry and Chemical Engineering, Xiamen University, Xiamen, Fujian 361005, China.

[3] Department of Nanoscience, Joint School of Nanoscience and Nanoengineering, University of North Carolina at Greensboro, Greensboro, NC 27401, United States.

E-mails: xuhuilin1224@gmail.com (XL); weiwu@xmu.edu.cn (WW); y_mo3@uncg.edu (YM)

Methodology

Block-localized wavefunction (BLW) method. Molecular orbital (MO) theory assumes that all electrons are in MOs which are delocalized to the whole system, whereas valance bond (VB) theory starts from localized atomic orbitals to build Lewis (resonance) structures and describes a conjugated system with several resonance structures.[1-3] Each resonance structure can be defined with a Heitler-London-Slater-Pauling (HLSP) function which is essentially a combination of a number of Slater determinants. To simplify the computational costs and combine the advantages of both MO and VB theories, we proposed the BLW method where a BLW corresponds to a unique electron-localized diabatic state (usually the most stable resonance state).[4-5] The fundamental assumption is that all electrons and primitive basis functions (χ) can be divided into k subgroups (blocks), and each MO is block-localized and expanded in only one block. Assuming that there are m_i basis and n_i electrons for block i, we can express block-localized MOs for this block as

$$\varphi_j^i = \sum_{\mu=1}^{m_i} C_{j\mu}^i \chi_\mu^i \tag{1}$$

Subsequently, the BLW for a closed-shell is defined using a Slater determinant as

$$\Psi^{\mathrm{BLW}} = \det\left|\left(\phi_1^1\right)^2\left(\phi_2^1\right)^2\cdots\left(\phi_{n_1/2}^1\right)^2\cdots\left(\phi_1^i\right)^2\cdots\left(\phi_{n_i/2}^i\right)^2\cdots\left(\phi_{/n_k/2}^k\right)^2\right| = \hat{A}\left[\Phi_1\Phi_2\cdots\Phi_k\right] \tag{2}$$

Orbitals in the same subspace are subject to the orthogonality constraint, but orbitals belonging to different subspaces are nonorthogonal. The BLW method is available at the DFT level with the geometry optimization and frequency computation capabilities.

Construction of BLWs for Pa_2B_2 For the studied Pa_2B_2, each atom is viewed as a block and thus pure atomic orbitals are used to construct strictly block-localized wavefunctions. Beside the core orbitals, the eight valence CMOs can be divided into three groups as we discussed above, *i.e.* four localized Pa-B σ orbitals, two delocalized σ and two delocalized π orbitals. In the delocalized states (Ψ_{del}), all eight CMOs are delocalized with contribution from all basis functions. To explore the electron delocalization effect of the above three kinds of CMOs, we constructed localized states step by step (see detail in Supporting Information). Firstly, the four localized Pa-B σ

orbitals are strictly localized on corresponding atoms and the remaining MOs are fully delocalized. Therefore, the energy difference (ΔE_{cov}) between such localized state Ψ_{loc}^{cov} and delocalized Ψ_{del} reflect the electron delocalization of the four Pa-B covalent bond. Secondly, we established strictly σ- and π-localized states (Ψ_{loc}^{σ} and Ψ_{loc}^{π}) by further localizing two σ and two π orbitals on two adjacent Pa and B atoms independently. Finally, all the eight CMOs are localized to construct total localized states (Ψ_{loc}^{tot}). As consequences, the energy difference between σ-, π- or total localized states and the Ψ_{loc}^{cov} represents the electron delocalization induced by σ, π orbitals and their total work. All the calculation are performed at VBSCF level and the delocalization energies are obtained at standard PBE0 and PBE0-DKH2 with HF electron densities.

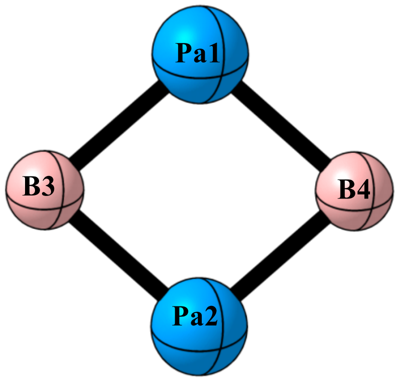

$$\Psi_{\rm del} = \hat{A}(\Omega_{\rm c}\phi^{1-4}_{\rm Pa_1\text{-}B_3}\phi^{1-4}_{\rm Pa_1\text{-}B_4}\phi^{1-4}_{\rm Pa_2\text{-}B_3}\phi^{1-4}_{\rm Pa_2\text{-}B_4}\bullet\phi^{1-4}_{\sigma_1}\phi^{1-4}_{\sigma_2}\bullet\phi^{1-4}_{\pi_1}\phi^{1-4}_{\pi_2})$$
$$\Psi^{\rm Cov}_{\rm Loc} = \hat{A}(\Omega_{\rm c}\phi^{1,3}_{\rm Pa_1\text{-}B_3}\phi^{1,4}_{\rm Pa_1\text{-}B_4}\phi^{2,3}_{\rm Pa_2\text{-}B_3}\phi^{2,4}_{\rm Pa_2\text{-}B_4}\bullet\phi^{1-4}_{\sigma_1}\phi^{1-4}_{\sigma_2}\bullet\phi^{1-4}_{\pi_1}\phi^{1-4}_{\pi_2})$$
$$\Psi^{\sigma}_{\rm Loc} = \hat{A}(\Omega_{\rm c}\phi\phi^{1,3}_{\rm Pa_1\text{-}B_3}\phi^{1,4}_{\rm Pa_1\text{-}B_4}\phi^{2,3}_{\rm Pa_2\text{-}B_3}\phi^{2,4}_{\rm Pa_2\text{-}B_4}\bullet\phi^{1,3}_{\sigma_1}\phi^{2,4}_{\sigma_2}\bullet\phi^{1-4}_{\pi_1}\phi^{1-4}_{\pi_2})$$
$$\Psi^{\pi}_{\rm Loc} = \hat{A}(\Omega_{\rm c}\phi^{1,3}_{\rm Pa_1\text{-}B_3}\phi^{1,4}_{\rm Pa_1\text{-}B_4}\phi^{2,3}_{\rm Pa_2\text{-}B_3}\phi^{2,4}_{\rm Pa_2\text{-}B_4}\bullet\phi^{1-4}_{\sigma_1}\phi^{1-4}_{\sigma_2}\bullet\phi^{1,3}_{\pi_1}\phi^{2,4}_{\pi_2})$$
$$\Psi^{\rm Tot}_{\rm Loc} = \hat{A}(\Omega_{\rm c}\phi^{1,3}_{\rm Pa_1\text{-}B_3}\phi^{1,4}_{\rm Pa_1\text{-}B_4}\phi^{2,3}_{\rm Pa_2\text{-}B_3}\phi^{2,4}_{\rm Pa_2\text{-}B_4}\bullet\phi^{1,3}_{\sigma_1}\phi^{2,4}_{\sigma_2}\bullet\phi^{1,3}_{\pi_1}\phi^{2,4}_{\pi_2})$$

Figure S1. The definition for the delocalized and various localized states, in which Ω_c represents the core orbitals. For molecular orbital ϕ, the superscripts refer to the basis functions from the specific atoms, while the subscripts mean the concerned bonds.

Alternatively, the basis functions for planar Pa_2B_2 can be divided σ and π components. Therefore, we are able to apply BLW method to re-construct Ψ_{loc}^{π} state by only localizing the π electrons but with all σ electrons delocalizing on the whole system

$$\Psi^{\pi}_{\rm Loc} = \hat{A}(\Omega_{\sigma}\phi_{\pi_{1,3}}\phi_{\pi_{2,4}}) \qquad (3)$$

where Ω_σ represents the delocalized σ orbitals, while $\pi_{1,3}$ and $\pi_{2,4}$ mean the π orbitals with two electrons localized on atoms 1and 3 and 2 and 4 respectively.It should be noted that we can't obtain the Ψ_{loc}^{σ} state with same strategy because the concerned delocalized σ orbitals share same Pa atomic orbitals with localized Pa-B σ orbitals.

Table S1. Orbital composition analysis by natural atomic orbital (NAO) method.

CMOs	Composition of atomic orbitals
HOMO	$11\%d_{xy}23\%f_{(s1+s3)}+15\%p_x$
HOMO-1	$15\%6d_{xy}17\%5f_{(s1)}+17\%p_y$
HOMO-2	$28\%6d_{z2}3\%f_{(0)}+5\%2s8\%2p_y$
HOMO-3	$11\%7s8\%d_{x2-y2}+27\%p_x$
HOMO-4	$19\%6d_{yz}9\%5f_{(s2)}+21\%p_z$
HOMO-5	$27\%d_{xz}5\%f_{(c1+c3)}+16\%p_z$
HOMO-6	$12\%7s3\%d_{x2-y2}+29\%2s$
HOMO-7	$7\%d_{xy}$ +38%2s

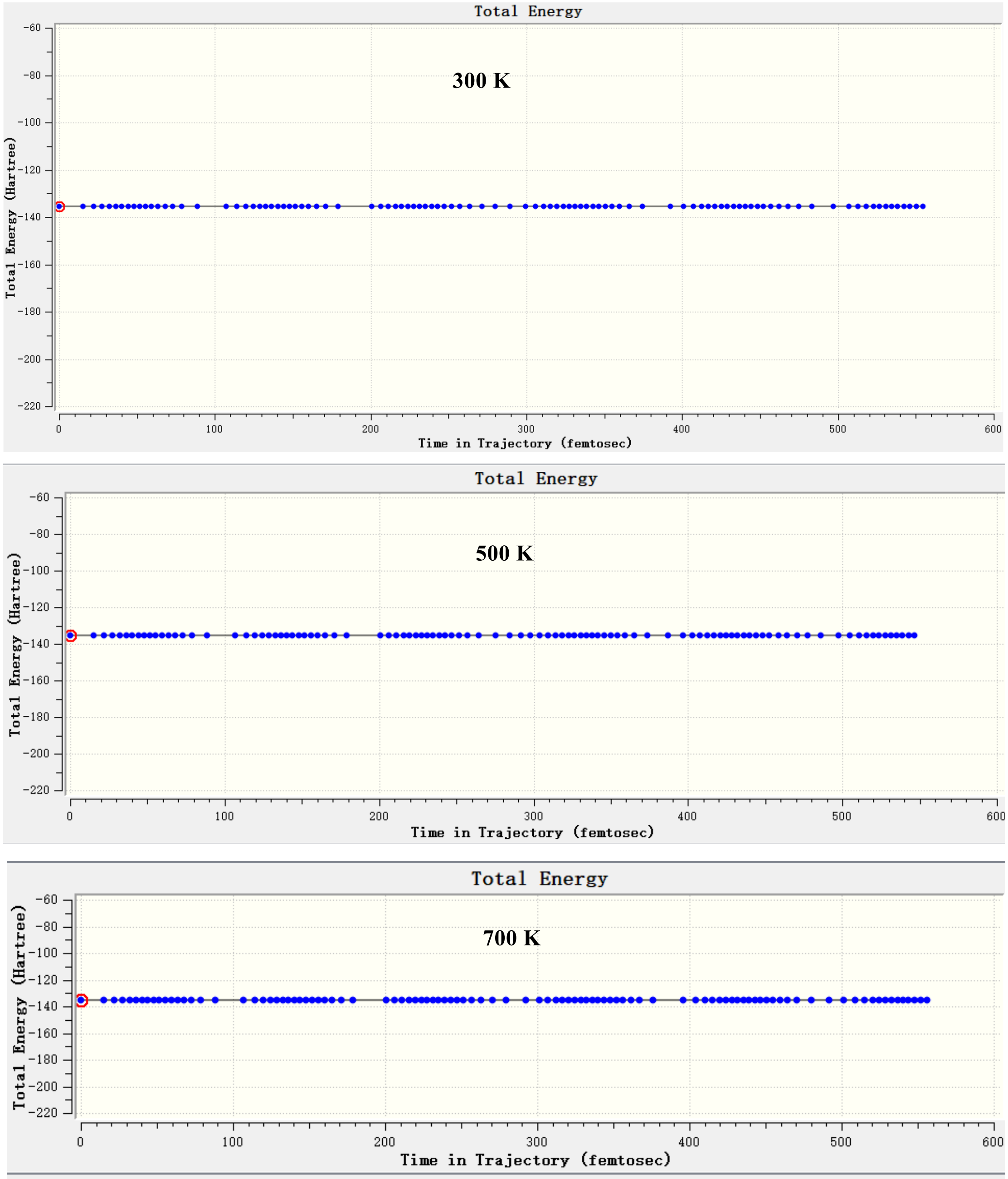

Figure S2. The change of total energy by BOMD at 300K, 500K and 700k.

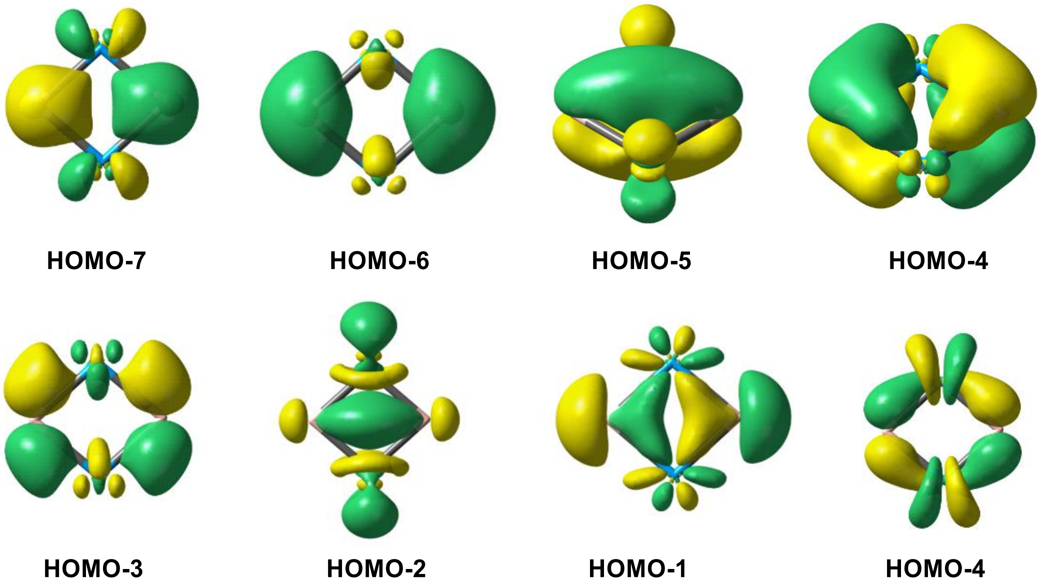

Figure S3. The eight CMOs at standard PBE0/ECP60MDF level.

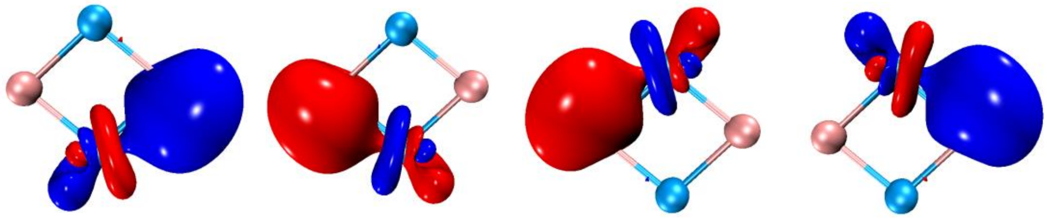

Four 2c-2e Pa-B orbitals (ON:1.91)

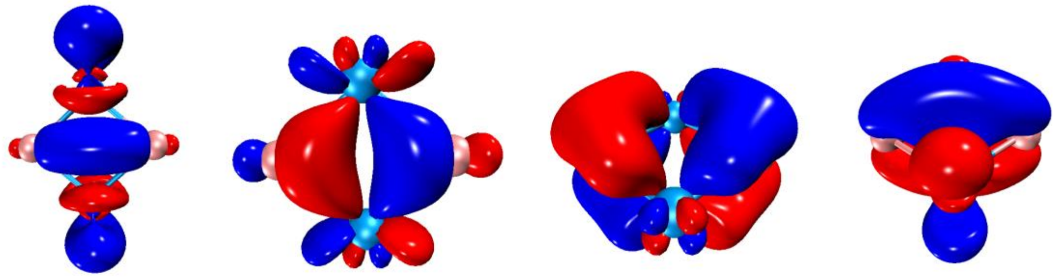

Two 4c-2e σ orbitals (ON:2.00) Two 4c-2e π orbitals (ON:2.00)

Figure S4. The AdNDP results at standard PBE0/ECP60MDF level.

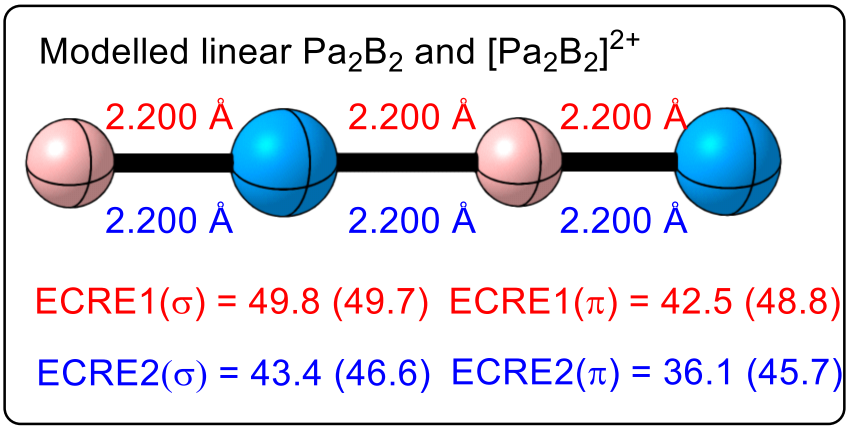

Figure S5. The evaluated extra cyclic resonance energy (ECRE, in kcal/mol) with modelled Pa_2B_2 (red data), $[Pa_2B_2]^{2+}$(blue data), where in which Pa-B bond distances are identical to that in the cyclic Pa2B2

The optimal geometries at PBE0/ECP60MDF level

Cyclic Pa_2B_2

Pa	0.00000000	1.43966810	0.00000000
Pa	0.00000000	-1.43966810	0.00000000
B	1.66325312	0.00000000	0.00000000
B	-1.66325312	0.00000000	0.00000000

π-localized Pa_2B_2 $\Psi^{\pi}_{\mathrm{Loc}}$

Pa	-0.0000000000	0.0767076846	1.5230106358
Pa	-0.0000000000	-0.0766991836	-1.5229991673
B	0.0000000000	1.6627553185	0.0677853056
B	0.0000000000	-1.6627638195	-0.0677967741

linear Pa_2B_2

Pa	0.00000000	0.00000000	-2.02564805
Pa	0.00000000	0.00000000	2.23637898
B	0.00000000	0.00000000	-4.05913053
B	0.00000000	0.00000000	0.22382772

linear $[Pa_2B_2]^{2+}$

Pa	0.00000000	0.00000000	-2.06718739
Pa	0.00000000	0.00000000	2.30378594
B	0.00000000	0.00000000	-4.34443711
B	0.00000000	0.00000000	0.03834349

References

(1) Cooper, D., *Valence bond theory*. Elsevier: 2002.

(2) Shaik, S. S.; Hiberty, P. C., *A chemist's guide to valence bond theory*. John Wiley & Sons: 2007.

(3) Wu, W.; Su, P.; Shaik, S.; Hiberty, P. C., Classical valence bond approach by modern methods. *Chem. Rev.* **2011,** *111* (11), 7557-7593.

(4) Mo, Y.; Peyerimhoff, S. D., Theoretical analysis of electronic delocalization. *J. Chem. Phys.* **1998,** *109* (5), 1687-1697.

(5) Mo, Y.; Song, L.; Lin, Y., Block-localized wavefunction (BLW) method at the density functional theory (DFT) level. *J. Phys. Chem. A* **2007,** *111* (34), 8291-8301.
